# Empagliflozin alleviates atherosclerotic calcification by inhibiting osteogenic differentiation of vascular smooth muscle cells

**DOI:** 10.3389/fphar.2023.1295463

**Published:** 2023-11-29

**Authors:** Junping Li, Changping Li, Zhaoqi Huang, Chunling Huang, Juanzhang Liu, Tao Wu, Shuwan Xu, Peibiao Mai, Dengfeng Geng, Shuxian Zhou, Kun Zhang, Zhaoyu Liu

**Affiliations:** ^1^ Department of Cardiology, Sun Yat-sen Memorial Hospital, Sun Yat-sen University, Guangzhou, China; ^2^ Department of Cardiology, Guangdong Provincial Key Laboratory of Arrhythmia and Electrophysiology, Sun Yat-Sen Memorial Hospital, Sun Yat-Sen University, Guangzhou, China; ^3^ Medical Research Center, Guangdong Provincial Key Laboratory of Malignant Tumor Epigenetics and Gene Regulation, Sun Yat-Sen Memorial Hospital, Sun Yat-Sen University, Guangzhou, China; ^4^ Department of Cardiology, The Eighth Affiliated Hospital, Sun Yat-sen University, Shenzhen, China

**Keywords:** atherosclerosis, arterial calcification, empagliflozin, osteogenic differentiation, vascular smooth muscle cell

## Abstract

SGLT-2 inhibitors, such as empagliflozin, have been shown to reduce the occurrence of cardiovascular events and delay the progression of atherosclerosis. However, its role in atherosclerotic calcification remains unclear. In this research, ApoE^−/−^ mice were fed with western diet and empagliflozin was added to the drinking water for 24 weeks. Empagliflozin treatment significantly alleviated arterial calcification assessed by alizarin red and von kossa staining in aortic roots and reduced the lipid levels, while had little effect on body weight and blood glucose levels in ApoE^−/−^ mice. *In vitro* studies, empagliflozin significantly inhibits calcification of primary vascular smooth muscle cells (VSMCs) and aortic rings induced by osteogenic media (OM) or inorganic phosphorus (Pi). RNA sequencing of VSMCs cultured in OM with or without empagliflozin showed that empagliflozin negatively regulated the osteogenic differentiation of VSMCs. And further studies confirmed that empagliflozin significantly inhibited osteogenic differentiation of VSMCs via qRT-PCR. Our study demonstrates that empagliflozin alleviates atherosclerotic calcification by inhibiting osteogenic differentiation of VSMCs, which addressed a critical need for the discovery of a drug-based therapeutic approach in the treatment of atherosclerotic calcification.

## 1 Introduction

Atherosclerotic plaque rupture can lead to acute myocardial infarction and stroke (Tomaniak et al., 2020). Atherosclerotic calcification (AC) is a pathological feature of advanced atherosclerosis (Dai et al., 2022). Especially, micro and fragmented calcifications in atherosclerotic plaques can increase the risk of plaque rupture (Jinnouchi et al., 2020). Vascular calcification has been considered as a passive and unregulated degenerative process in the past (Shioi and Ikari, 2018). However, accumulating studies have shown that vascular calcification is a tightly regulated and active process (Johnson et al., 2006; Durham et al., 2018). Detailed histological analyses of human coronary atherosclerotic calcification size and location have implicated VSMCs as major cellular orchestrators of atherosclerotic calcification (Durham et al., 2018). Numerous studies showed that osteogenic differentiation of VSMCs plays an important role in the formation of atherosclerotic calcification (Malhotra et al., 2019; Dai et al., 2022; Woo et al., 2023a). However, no drugs can effectively prevent the occurrence and progression of atherosclerotic calcification in clinic so far.

Empagliflozin (EMPA), a sodium-glucose cotransporter 2 inhibitor (SGLT2i), is a new oral drug for the treatment of type 2 diabetes mellitus (T2DM). Numerous studies showed that empagliflozin exerted cardiovascular protective effects, such as inhibiting the proliferation and migration of human aortic smooth muscle cells (Sukhanov et al., 2021), preventing neointima formation (Takahashi et al., 2019; Dutzmann et al., 2022), inhibiting the formation of abdominal aortic aneurysm (Ortega et al., 2019), and reducing arterial stiffness in diabetic patients (Cherney et al., 2014; Chilton et al., 2015; Bosch et al., 2019). Moreover, empagliflozin can attenuate atherosclerotic plaque formation and delay the progression of atherosclerosis in both diabetic and nondiabetic mice (Pennig et al., 2019; Liu et al., 2021a; Liu et al., 2021b). However, the effect of SGLT2i on atherosclerotic calcification remains unclear.

In this study, we aimed to explore the effects of empagliflozin on atherosclerotic calcification in ApoE −/− mice fed with a western diet and to identify the underlying mechanism. We found that empagliflozin alleviated atherosclerotic calcification *in vivo* and inhibited calcification of primary VSMCs and aortic rings *in vitro*. Mechanistically, empagliflozin negatively regulates the osteogenic differentiation of VSMCs.

## 2 Materials and methods

### 2.1 Animal experiments

The animal experiment was conducted with the approval of the Animal Experiment Ethics Committee of Sun Yat-sen University (Approval NO. AP20220195). And the animals were kept in the Laboratory Animal Center of Sun Yat-sen University East Campus under a constant temperature of 25°C and a 12-h light-dark cycle. The atherosclerotic calcification (AC) model was established according to previous study (Cai et al., 2016; Ouyang et al., 2022). In brief, eighteen 9-week-old male ApoE^−/−^ mice were randomly divided into two groups: AC group (*n* = 9) and AC plus EMPA group (*n* = 9). Mice in both groups were fed Western diet (0% cholesterol, 40 kcal% fat, 17 kcal% protein and 43 kcal% carbohydrate; HFHC, Diets) for 24 weeks. The AC group was given normal drinking water, and the AC plus EMPA group was treated with empagliflozin (30 mg/kg/d, S31680, Yuanye Biological Co., Shanghai, China) in drinking water. Drinking water was changed every 3 days.

### 2.2 Preparation of frozen sections of aortic roots

The aortic roots were fixed in 4% paraformaldehyde for 20 min and dehydrated in 30% sucrose solution overnight. And then the aortic roots were embedded with OCT and made into 8 μm frozen sections. The section direction was kept perpendicular to the direction of the aortic root, and the sections were collected when the three valves of the heart were in the same plane. Serial cryosections were collected on different slides, and samples from five different layers were placed in each section.

### 2.3 Hematoxylin-eosin staining and oil red O staining

Hematoxylin-eosin staining was used to evaluate the histopathological changes according to the instructions of the kit (BASO, BA4097 and Solarbio, G1100). Oil red O staining was used to assess atherosclerotic plaque formation according to the provided protocol of the kit (G1015, Servicebio).

### 2.4 Alizarin red staining and quantitative analysis

Serial cryosections were collected on different slides, and samples from five different layers were placed in each section. Frozen sections were fixed in 4% paraformaldehyde for 30 min and washed with PBS for 5 min*3 times, then immersed in 2% alizarin red staining solution (PH4.2) (G1038, Servicebio) for 5 min. The areas of positive staining are orange-red. Quantitative analysis of calcium deposit by measuring the gray value of calcified areas with ImageJ software, the mean positive area of these five different layers was taken as one experimental data for each mouse. VSMCs were washed with PBS for 3 times, then immersed in 2% alizarin red staining solution (PH4.2) (G1038, Servicebio) for 2 min, following washed with PBS for 5min*5 times. After alizarin red staining of VSMCs, the cells were decalcified with 10% cetylpyridine chloride (CPC) and diluted 3 times with PBS, and then the OD value was detected by microplate reader at a wavelength of 562 nm.

### 2.5 Von kossa staining and quantitative analysis

Serial cryosections were collected on different slides, and samples from five different layers were placed in each section. Frozen sections were immersed in deionized water for 2 min to remove OCT, then stained with von Kossa staining solution (G1043, Servicebio) to cover the tissues and irradiated under ultraviolet light for 4 h. The area of calcium salt deposition is black. Quantitative analysis of calcium deposit by measuring the gray value of calcified areas with ImageJ software. The mean positive area of these five different layers was taken as one experimental data for each mouse.

### 2.6 Quantification of atherosclerotic lesion area and calcification area

ImageJ software was used to evaluate the atherosclerotic plaque area according to the results of oil red O staining, and ImageJ software was used to evaluate the calcification area according to the results of alizarin red staining and von kossa staining. Alizarin red staining or von kossa staining positive area/plaque area = calcification area. The ratio of the oil red O staining positive area to the vascular ring area was calculated as the proportion of plaque area. The ratio of alizarin red staining or von kossa staining positive area to the plaque area was calculated as the proportion of calcification area. The average of five samples on each section was taken as one data.

### 2.7 Serum cholesterol and triglycerides detection

Total cholesterol assay kit (A111-1-1) and triglycerides assay kit (A110-1-1) were bought from Nanjing Jiancheng Bioengineering Institute of China. The serum total cholesterol and triglycerides were determined according to the manufacturer’s instructions.

### 2.8 Serum calcium, phosphorus and creatinine detection

Serum calcium assay kit (C004-2) and creatinine assay kit (C011-2) were bought from Nanjing Jiancheng Bioengineering Institute of China. Phosphorus (pi) colorimetric assay kit (E-BC-K245-M) was bought from Elabscience. All indicators were detected was performed strictly according to the manufacturer’s instructions.

### 2.9 Calcium content detection and alkaline phosphatase (ALP) activity

Calcium assay kit (C004-2) and alkaline phosphatase (ALP) assay kit (A059-2) were bought from Nanjing Jiancheng Bioengineering Institute of China. The ALP activity and calcium content were determined according to the manufacturer’s instructions. The results were then normalized by the protein content determined by the BCA protein assay kit (K3000) from Biocolor BioScience of Shanghai, China.

### 2.10 Isolation, culture and calcification stimulation of primary cells

Primary rat thoracic aorta vascular smooth muscle cells (VSMCs) were extracted and cultured. Briefly, Male rats weighing about 200 g were killed by deep anesthesia with isoflurane (5%). After routine disinfection, the thoracic cavity was opened, and the thoracic aorta was blunt-separated into complete media (CM), which is defined as high-glucose Dulbecco’s modified Eagle’s medium (DMEM) supplemented with 10% fetal bovine serum (FBS) and 1% penicillin/streptomycin (P/S). After stripping the adventitia and intima of aorta under a microscope, the media of aorta was cut into fragments as much as possible and attached to the T25 culture bottle. After 6–7 h, the cells were added with DMEM (containing 20% FBS plus 1% P/S), and the cells could be passaged at about 7–8 days. In this study, VSMCs from passage 5 to passage 9 were used in DMEM containing 10% FBS and 1% P/S. VSMCs calcification was induced by osteogenic media (OM) (DMEM media with 10% FBS and 1% P/S, supplemented with β-glycerophosphate disodium salt (β-GP, Sigma-Aldrich, G9422) (10 mM) and CaCl2 (Sigma-Aldrich, C5670) (3.5 mM)) For 5–7 days. For inorganic phosphate (Pi) - induced VSMCs calcification, its working concentration is 2.6 mM and VSMCs were treated for 5–7 days.

### 2.11 Isolation and culture of aortic rings

Thoracic aorta of 9-week-old male C57BL/6 mice were cut into approximately 2 mm rings, cultured in osteogenic media (OM) (10 mM β-GP plus 3.5 mM CaCl2) or 2.6 mM Pi, and the experimental groups treated with empagliflozin (1 μM) for 12 days. And then they were embedded in OCT, cut into 6–8 μm and attached to slides for Alizarin red staining and von Kossa staining.

### 2.12 RNA-seq analysis

Transcriptome sequencing experiments were performed by Guangzhou Ige BIOTECHNOLOGY Co., Ltd. Briefly, RNA-seq transcriptome library was sequenced with the Illumina HiSeq 2000 (2x 100 bp read length). Differentially expressed genes with adjusted *p*-value ≤0.05 were identified. And then Gene Ontology (GO) enrichment analysis was performed using DAVID.

### 2.13 Quantitative real time polymerase chain reaction (qRT-PCR)

Total RNA was extracted from treated VSMCs using TRIzol reagent (Invitrogen, Waltham, MA). Extracted mRNA was reverse transcribed into cDNA using Hifair^®^ II 1st Strand cDNA Synthesis Kit (Yeasen, Shanghai, China). Individual quantitative RT-PCR was performed using gene-specific primers as shown in Supplementary Table S1.

### 2.14 Statistical analysis

All data were showed as means ± SEM. Differences of data between the two groups were compared by unpaired Student’s t-test. Differences of data among multiple groups were determined by one-way ANOVA followed by the LSD test or Tamhane test. Statistical analysis was performed with SPSS version 24, and *p* < 0.05 was considered statistically significant. GraphPad Prism 8.3 was used to draw figures.

## 3 Results

### 3.1 Empagliflozin reduced lipid levels but had no effect on body weight and blood glucose level in non-diabetic atherosclerosis mouse model

**FIGURE 1:**
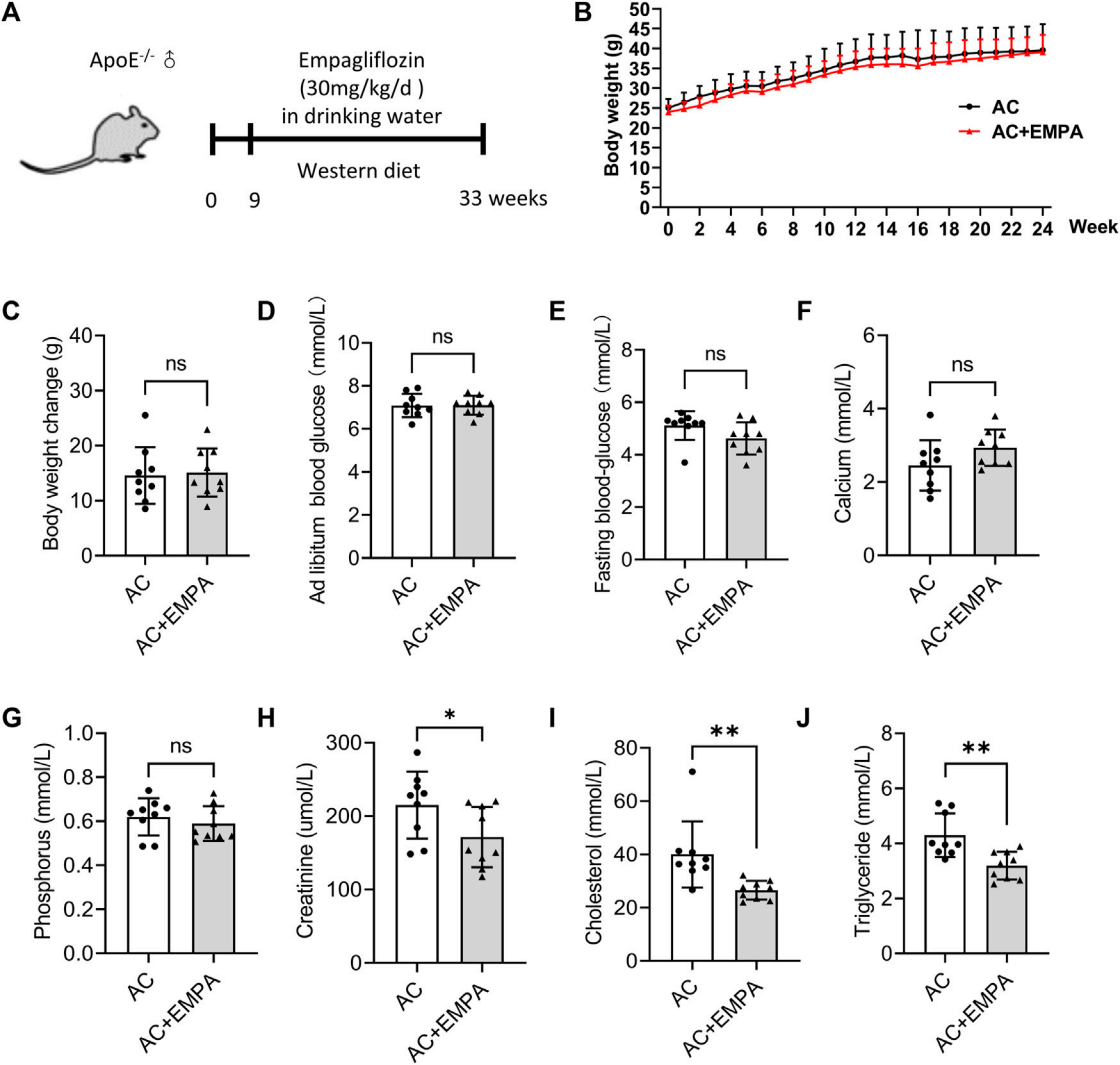
Empagliflozin reduced lipid levels but had no effect on blood glucose in mouse atherosclerotic calcification (AC). ApoE^−/−^ mice were fed with western diet with or without empagliflozin (EMPA) for 24 weeks to induce atherosclerotic calcification (AC). **(A)** Experimental design scheme. **(B)** The changes of body weight weekly during the experiment. **(C)** Comparison of weight gain (final body weight minus initial body weight) during the experiment. *Ad libitum* blood glucose **(D)** and fasting blood glucose **(E)** of mice in both groups on the day before the end of the experiment. Serum calcium **(F)**, phosphorus **(G)**, creatinine levels **(H)**, total cholesterol **(I)** and triglyceride **(J)** levels in both groups at the end of the experiment. ns means no statistical differences. *p < 0.05, **p < 0.01.

To investigate the effect of empagliflozin on atherosclerotic calcification (AC) *in vivo*, ApoE^−/−^ mice were fed with western diet and empagliflozin was added to the drinking water for 24 weeks (Figure 1A). Because obesity, diabetes mellitus, and hyperlipidemia are crucial risk factors of atherosclerosis (Jayachandran and Qu, 2021), metabolic parameters, including body weight, blood glucose, serum total cholesterol, and serum triglycerides were detected in ApoE^−/−^ mice with atherosclerosis calcification. Over the 24 weeks period, empagliflozin did not affect mice body weight (Figures 1B, C). After 24 weeks feeding, empagliflozin treatment did not affect *ad libitum* blood glucose level (7.10 ± 0.15 mmol/L in the AC plus EMPA group vs. 7.09 ± 0.18 mmol/L in the AC group, *p* = 0.962) and fasting blood glucose level (4.62 ± 0.20 mmol/L in the AC plus EMPA group vs. 5.11 ± 0.18 mmol/L in the AC group, *p* = 0.094) in these non-diabetic mice (Figures 1D, E). Empagliflozin treatment did not affect serum calcium (2.93 ± 0.49 mmol/L in the AC plus EMPA group vs. 2.45 ± 0.69 mmol/L in the AC group, *p* = 0.1057) and phosphorus levels (0.59 ± 0.79 mmol/L in the AC plus EMPA group vs. 0.62 ± 0.85 mmol/L in the AC group, *p* = 0.45) in these non-diabetic mice (Figures 1F–H). However, empagliflozin reduced the levels of total cholesterol (26.56 ± 1.19 mmol/L in the AC plus EMPA group vs. 40.01 ± 4.14 mmol/L in the AC group, *p* = 0.007, Figure 1I) and triglyceride (3.19 ± 0.17 mmol/L in the AC plus EMPA group vs. 4.29 ± 0.26 mmol/L in the AC group, *p* = 0.003, Figure 1J). Empagliflozin reduced the serum creatinine level (171.41 ± 10.97 μmol/L in the AC plus EMPA group vs. 215.10 ± 45.62 μmol/L in the AC group, *p* = 0.048). These data suggested that empagliflozin reduced lipid and creatinine levels but it had no effect on blood glucose, calcium, phosphorus in non-diabetic mouse atherosclerosis model.

### 3.2 Empagliflozin alleviated arterial calcification during atherosclerosis

**FIGURE 2:**
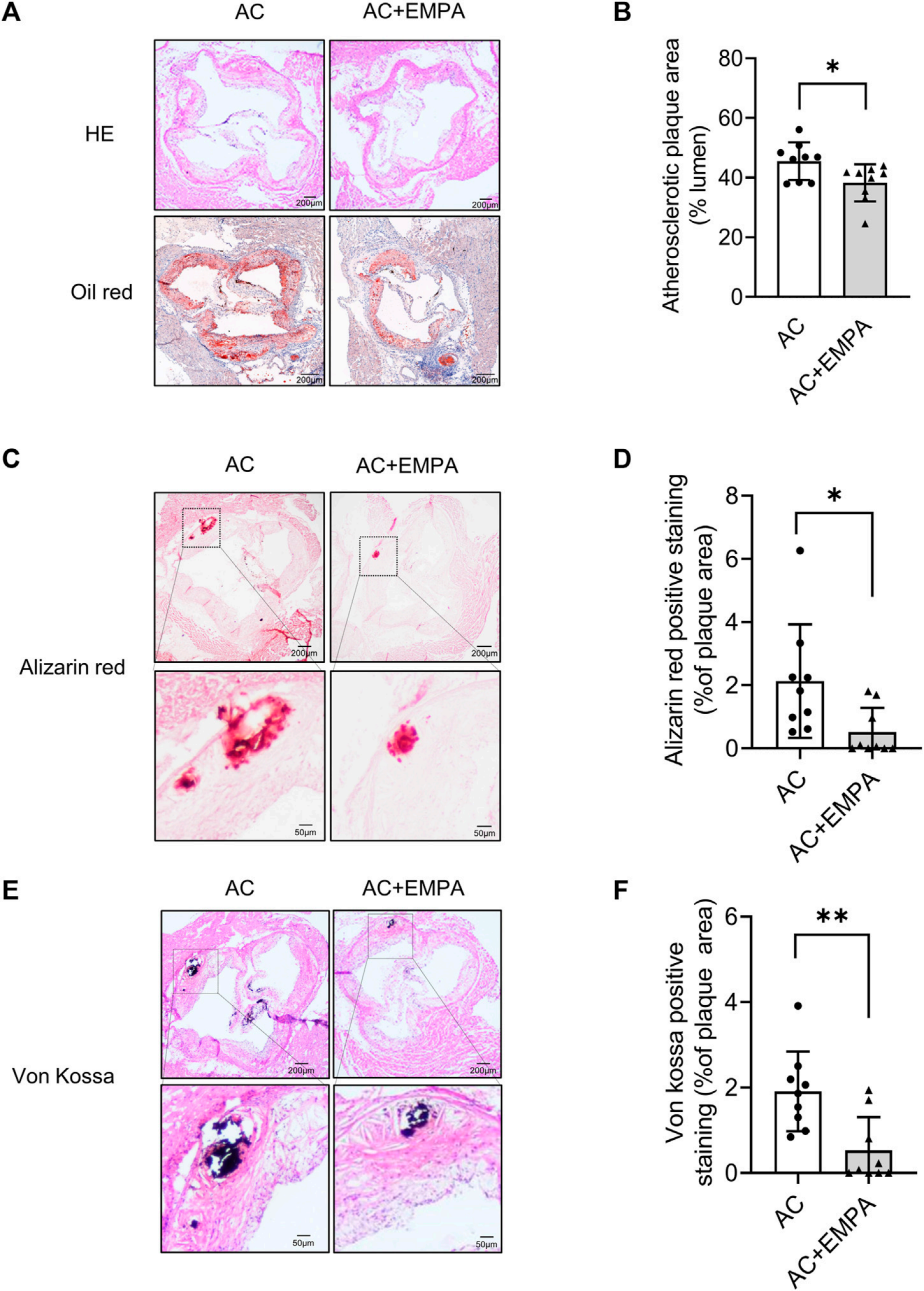
Empagliflozin alleviated western diet - induced atherosclerotic lesion area and calcification area *in vivo*. ApoE^−/−^ mice were fed with western diet with or without empagliflozin (EMPA) for 24 weeks to induce atherosclerotic calcification (AC). **(A)** Representative images of hematoxylin-eosin (HE) staining and oil red O staining of aortic roots of mice in indicated groups. **(B)** Quantitative analysis of atherosclerotic plaque area based on oil red O staining. **(C)** Representative images of alizarin red staining in atherosclerotic lesion areas of aortic roots. **(D)** Quantitative analysis of alizarin red staining positive area (alizarin red staining positive area/atherosclerotic plaque area). **(E)** Representative images of von Kossa staining in atherosclerotic lesion areas of aortic roots. **(F)** Quantitative analysis of von Kossa staining positive area (von Kossa staining positive area/atherosclerotic plaque area). *p < 0.05, **p < 0.01.

We next detected arterial calcification in these ApoE^−/−^ mice. As shown in Figures 2A, B, empagliflozin treatment reduced atherosclerotic plaque area in the aortic roots. Importantly, empagliflozin treatment significantly inhibited calcium nodule formation in atherosclerotic lesion areas as assessed by alizarin red (Figures 2C, D) and von Kossa staining (Figures 2E, F). These results suggested that empagliflozin alleviated western diet - induced atherosclerotic calcification *in vivo*.

### 3.3 Empagliflozin inhibited calcification of primary VSMCs *in vitro*

**FIGURE 3:**
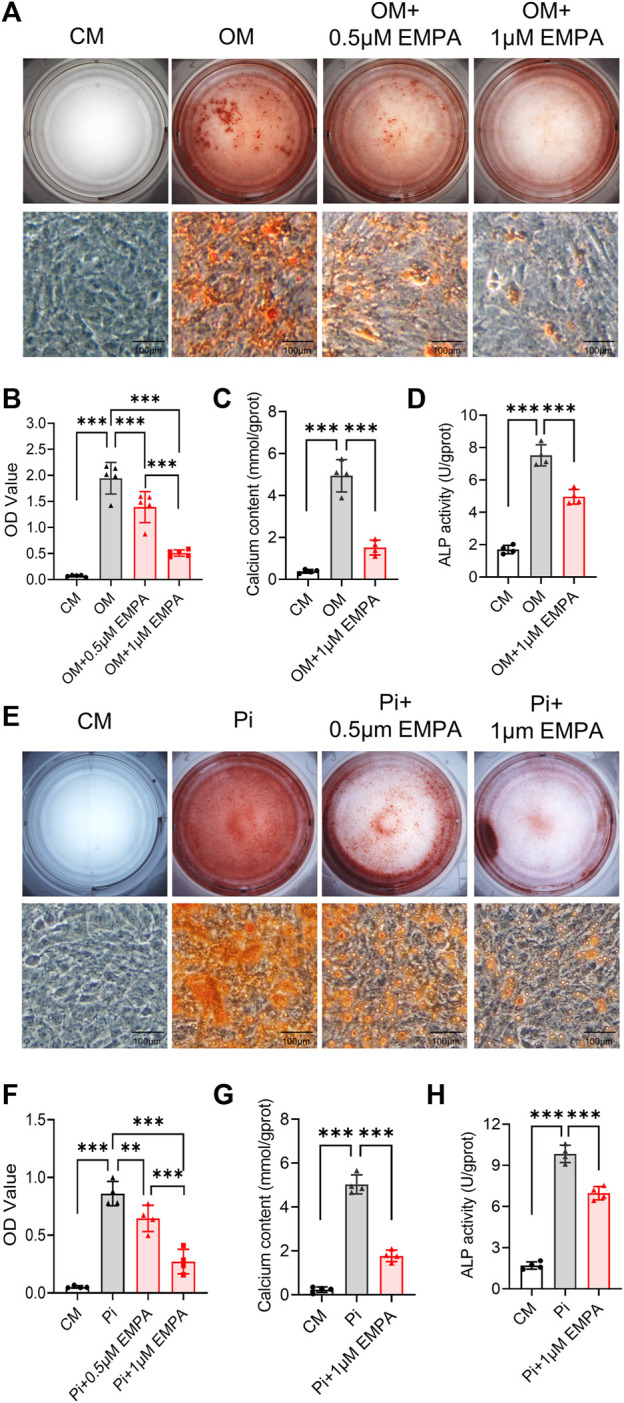
Empagliflozin inhibited calcification of primary VSMCs *in vitro*. Primary VSMCs were incubated in CM, or OM **(A–D)** or Pi (2.6 mM) **(E–H)** with or without EMPA (1 μM) for 5–7 days *in vitro*. Alizarin red staining was used to evaluate cell calcification. **(A)** Representative images of alizarin red staining for calcium nodule formation of primary VSMCs treated with CM or OM with or without EMPA (*n* = 5), scale bar = 100 μm. **(B)** Quantitative analysis of calcium deposit by a microplate reader at a wavelength of 562 nm (*n* = 5). **(C)** Quantitative analysis of calcium content with a calcium assay Ki (*n* = 4). **(D)** Quantitative analysis of ALP activity with an ALP assay Kit (*n* = 4). **(E)** Representative images of Alizarin red staining for calcium nodule formation of primary VSMCs treated with CM or Pi with or without EMPA (*n* = 4), scale bar = 100 μm. **(F)** Quantitative analysis of calcium deposit by a microplate reader at a wavelength of 562 nm (*n* = 4). **(G)** Quantitative analysis of calcium content with a calcium assay kit (*n* = 4). **(H)** Quantitative analysis of ALP activity with an ALP assay kit (*n* = 4). **p < 0.01, ***p < 0.001. ALP, alkaline phosphatase; CM, complete media; EMPA, empagliflozin; OM, osteogenic media; Pi, inorganic phosphate; VSMCs, vascular smooth muscle cells.

Vascular smooth muscle cells (VSMCs) are the key cell type involved in atherosclerotic calcification (Yang et al., 2020). We next investigate VSMCs calcification induced by osteogenic media (OM) and inorganic phosphate (Pi) *in vitro*. The results showed that 0.5 and 1 μM empagliflozin inhibited calcification of primary VSMCs induced by OM or Pi. Alizarin red staining evaluation showed that the inhibitory effect of 1 μM empagliflozin was more significant (Figures 3A, B, E, F). Moreover, 1 μM empagliflozin exhibited significantly decreased calcium content and ALP activity induced by OM or Pi in VSMCs calcification (Figures 3C, D, G, H). These results indicated that empagliflozin inhibited calcification of primary VSMCs *in vitro*.

### 3.4 Empagliflozin inhibited calcification of aortic rings *ex vivo*

**FIGURE 4:**
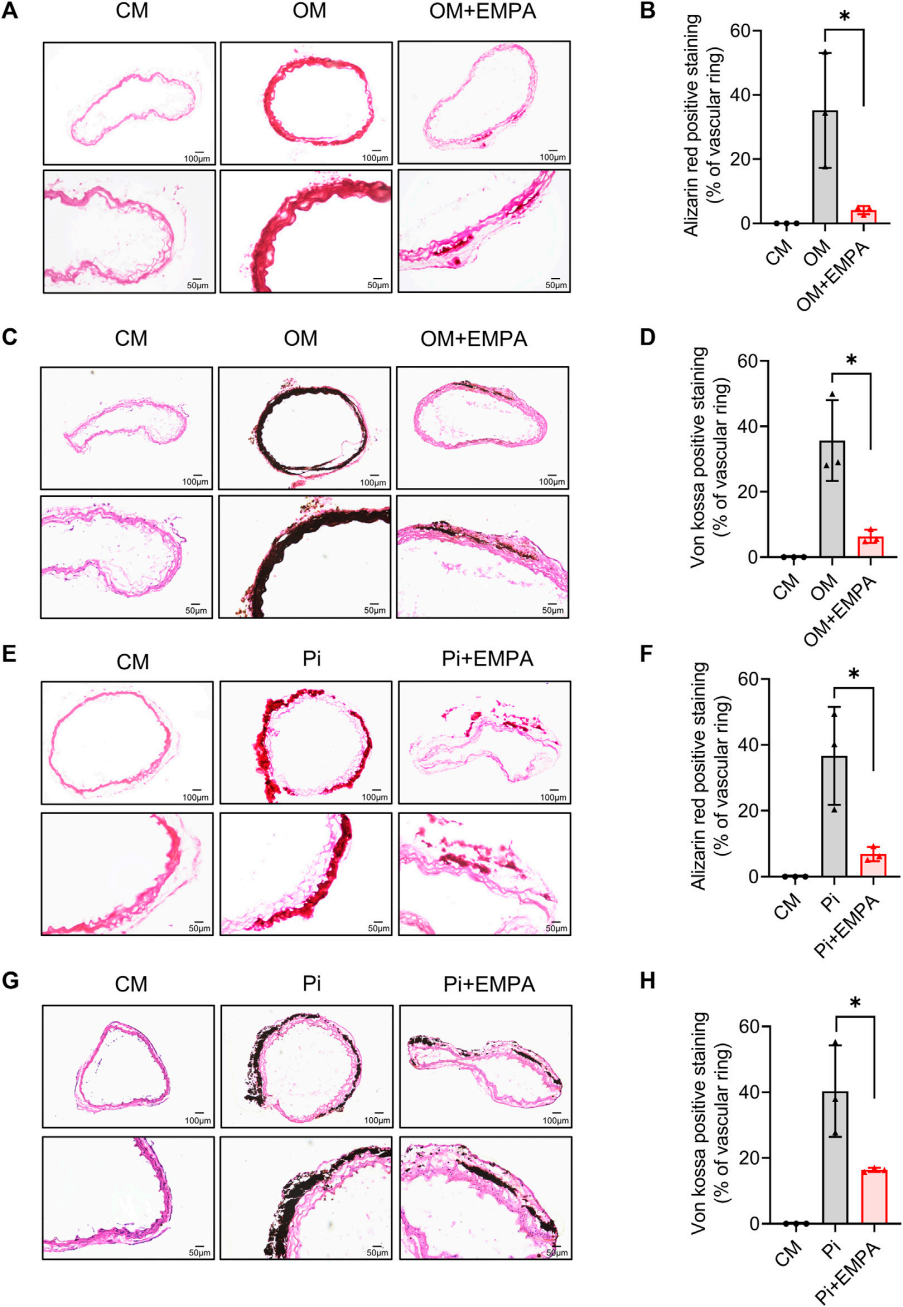
Empagliflozin alleviated calcification of aortic ring *ex vivo*. Thoracic aorta rings of 9-week-old male C57BL/6 mice were incubated in CM or OM **(A–D)** or Pi (2.6 mM) **(E–H)** with or without EMPA (1 μM) for 12 days *in vitro*. Alizarin red staining and von kossa staining was used to evaluate cell calcification. Representative images of alizarin red staining **(A)** and von kossa staining **(C)** for calcium nodule formation of aortic ring treated with CM or OM with or without EMPA (*n* = 3), Scale bar = 50 μm. Quantitative analysis of calcium deposit of figure A **(B)** and figure C **(D)** by measuring the gray value of calcified areas with ImageJ software (*n* = 3). Representative images of alizarin red staining **(E)** and von kossa staining **(G)** for calcium nodule formation of aortic ring treated with CM or Pi with or without EMPA (*n* = 3), scale bar = 50 μm. Quantitative analysis of calcium deposit of figure E **(F)** and figure G **(H)** by measuring the gray value of calcified areas with ImageJ software (*n* = 3). *p < 0.05. CM, complete media; EMPA, empagliflozin; OM, osteogenic media; Pi, inorganic phosphate.

To further verify the inhibitory effect of empagliflozin on VSMCs calcification, we next investigated the effect of empagliflozin on aortic rings calcification induced by osteogenic media (OM) or inorganic phosphate (Pi) *in vitro*. Alizarin red staining and von kossa staining were used to assess calcium deposition. The results showed that empagliflozin significantly inhibited calcium deposition in aortic rings induced by OM (Figures 4A–D). Similar results were observed in Pi - induced calcification of the aortic rings (Figures 4E–H). These results indicated that empagliflozin inhibited vascular calcification of aortic rings *ex vivo*.

### 3.5 Empagliflozin negatively regulated the osteogenic differentiation of VSMCs

**FIGURE 5:**
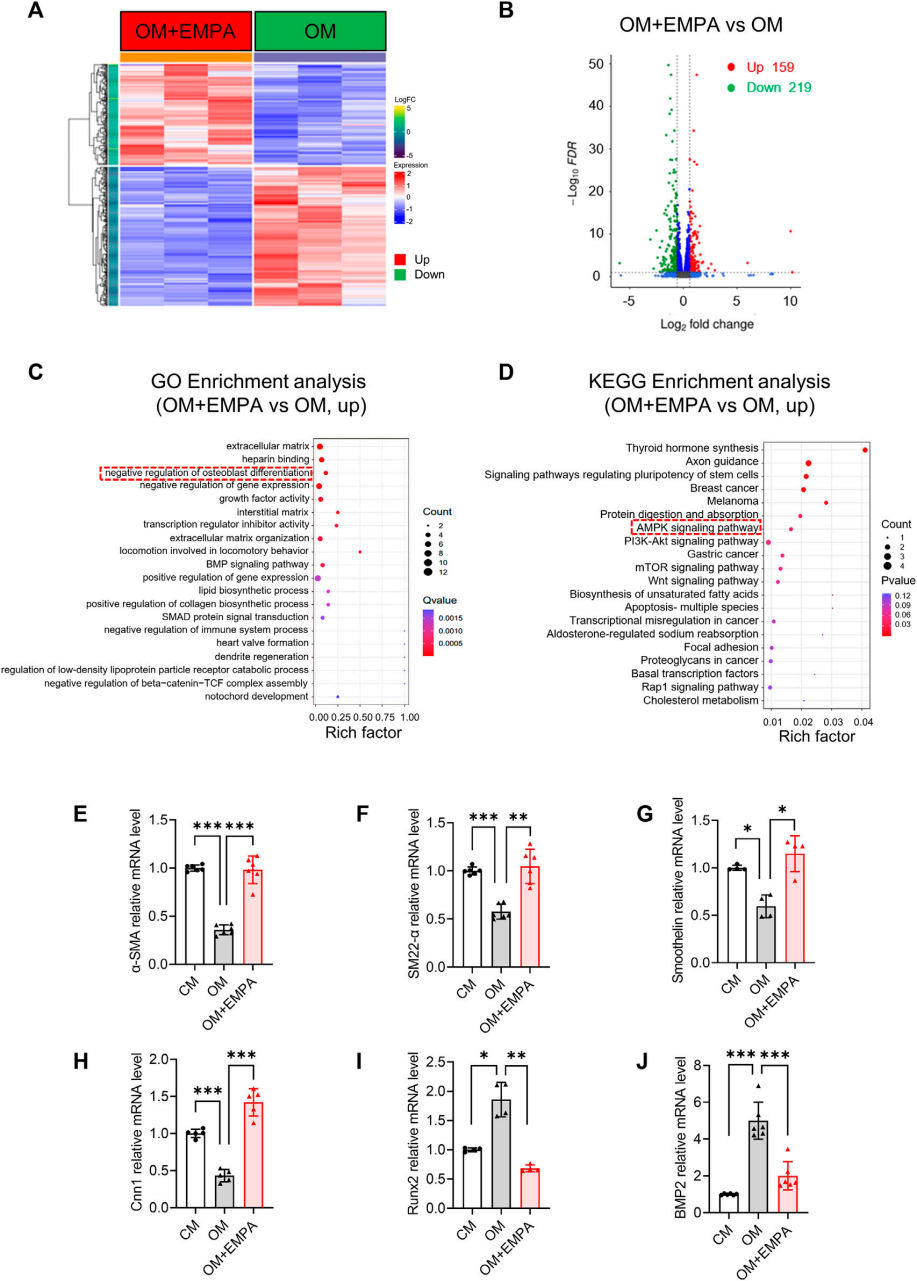
Empagliflozin negatively regulates the osteogenic differentiation of VSMCs. **(A–C)** Primary VSMCs were incubated in OM with or without EMPA (1 μM) for 4 days *in vitro* (*n* = 3). **(A)** Heatmap of differentially expressed genes (DEGs) from RNA-seq data. **(B)** Volcano plot from RNA-seq data. **(C)** GO enrichment analysis for the DEGs. **(D)** KEGG enrichment analysis for the DEGs. **(E–J)** Primary VSMCs were incubated in OM with or without EMPA (1 μM) for 7 days *in vitro* (*n* = 4–6). The mRNA levels of α-SMA **(E)**, SM22-α **(F)**, Smoothelin **(G)**, Cnn1**(H)**, Runx2 **(I)** and BMP2 **(J)** was detected by qRT-PCR. *p < 0.05, **p < 0.01, ***p < 0.001. α-SMA: alpha-smooth muscle actin; BMP2, Bone morphogenetic protein 2; Cnn1, calponin 1; EMPA, empagliflozin; KEGG, Kyoto Encyclopedia of Genes and Genomes. OM, osteogenic media; Pi, inorganic phosphate; Runx2, runt-related transcription factor 2; SM22-α, smooth muscle 22 alpha; VSMCs, vascular smooth muscle cells.

To determine how empagliflozin exerts its anti-vascular calcification effect, the VSMCs RNA-seq analysis was performed. As shown in Figure 5A, VSMCs cultured in OM with empagliflozin showed a significantly difference in gene expression compared with OM group. A total of 378 genes were differentially expressed in VSMCs treated with empagliflozin, including 159 upregulated genes and 219 downregulated genes (Figure 5B). The 159 upregulated genes were undergone GO enrichment analysis. As shown in Figure 5C, the enriched items were clustered in extracellular matrix, negative regulation of cell migration and negative regulation of osteoblast differentiation. Moreover, KEGG enrichment analysis showed that empagliflozin can activate the AMPK signaling pathway (Figure 5D). Accumulating studies have shown that osteogenic differentiation of VSMCs plays a crucial role in the process of vascular calcification (Leopold, 2015; Durham et al., 2018; Yang et al., 2020; Sutton et al., 2023). Then we further verify whether empagliflozin inhibits VSMCs calcification via negatively regulating the osteogenic differentiation of VSMCs. We detected the changes of VSMCs contractile phenotype markers (α-SMA, SM22-α, Cnn1 and Smoothelin) and VSMCs osteogenic differentiation transcription factor Runx2 and transforming growth factor BMP2 during VSMCs osteogenic differentiation by qRT-PCR. Compared with the CM group, the results showed that the VSMCs cultured in OM significantly induced the VSMCs from contractile phenotype to osteogenic phenotype, as indicated by the decreased mRNA levels of α-SMA, SM22-α, Cnn1 and Smoothelin and the increased mRNA levels of Runx2 and BMP2 (Figures 5E–J). However, empagliflozin reversed their mRNA levels of the markers described above (Figures 5E–J). These results suggested that empagliflozin inhibited the osteogenic differentiation of primary VSMCs *in vitro*.

## 4 Discussion

In the present study, we found that empagliflozin significantly alleviated atherosclerotic calcification in ApoE^−/−^ mice fed with western diet, and empagliflozin inhibited calcification of primary VSMCs and calcification of aortic rings *ex vivo*. In terms of mechanisms, empagliflozin inhibits vascular calcification by negatively regulating osteogenic differentiation of VSMCs.

In clinic, patients with high coronary calcium score have significantly increased mortality (Zeng et al., 2021). Due to lacking of effective drug intervention, exploring the etiology of calcification and finding the drugs for the prevention and treatment of vascular calcification have been a research hotspot in the cardiovascular field. Previous study has shown that empagliflozin could improve cardiovascular outcomes and reduce the incidence of death from any cause in patients with type 2 diabetes who were at high risk for cardiovascular events (Zinman et al., 2015). And empagliflozin could reduce the risk of death from cardiovascular causes or hospitalization for heart failure among patients with chronic heart failure, regardless of the presence or absence of diabetes (Packer et al., 2020). Previous study has suggested that empagliflozin can attenuate chronic kidney disease-induced vascular calcification (Lu et al., 2023). Here we found that empagliflozin can inhibit atherosclerotic calcification. These studies suggested that empagliflozin may be a potential drug in prevention and treatment of arterial calcification. Moreover, our data showed that empagliflozin did not reduce blood glucose level in non-diabetic atherosclerosis mouse model, suggesting the safety of empagliflozin in nondiabetic patients with atherosclerotic calcification.

Previous study has shown that atherosclerosis calcification is a process similar to bone formation and proceeds under the control of complex enzymatic and cellular pathways (Alexopoulos and Raggi, 2009). VSMCs undergo phenotypic change when vascular homeostasis changes due to injury or cytokine stimulation, and then migrate into the atherosclerotic intima to promote the progression of atherosclerosis (Nakagawa et al., 2010). During atherosclerotic plaque progression, VSMCs differentiate into early osteoblasts, which promote initial calcium deposition within the necrotic core of the lesion and form microcalcifications that may be associated with plaque rupture (Shioi and Ikari, 2018). Osteogenic differentiation of VSMCs has been extensively studied in atherosclerotic calcification and studies have shown that osteogenic differentiation of VSMCs are the main contributor in the process of atherosclerotic calcification (Woo et al., 2023b). During the osteogenic differentiation of VSMCs, the expression of VSMCs contractile phenotypic markers (such as α-SMA, SM22-α, Smoothelin and Cnn1) are significantly decreased, while the expression of bone and cartilage related transcription factors such as MSX2 and RUNX2 is increased (Nakagawa et al., 2010; Woo et al., 2023a). Moreover, VSMCs participates in a variety of atherogenic process signals and differentiation into Osteochondrogenic cells mainly through BMP signaling pathway (Nakagawa et al., 2010; Woo et al., 2023b), in which the expression of pro-osteogenic transforming growth factor BMP2 is significantly increased (Nakagawa et al., 2010). Studies have shown that Runx2 is low expressed in normal aorta, but highly expressed in VSMCs during atherosclerotic calcification and osteogenic differentiation *in vivo*, (Cai et al., 2016), and Runx2 can induce the expression of ALP activity to promote the osteogenic differentiation of VSMCs (Cai et al., 2016). Our data showed that empagliflozin significantly decreased the mRNA levels of Runx2 and BMP2 and ALP activity and increased the mRNA levels of α-SMA, SM22-α, Smoothelin and Cnn1 in primary VSMCs induced by osteogenic media *in vitro*, and that empagliflozin significantly inhibited calcification of aortic ring *ex vivo*, which may be the main mechanism by which empagliflozin inhibited atherosclerotic calcification. We further analyzed RNA sequencing results and found that empagliflozin can activate AMPK signaling pathway. Previous studies have shown that AMPK activation can inhibit VSMC calcification (Ouchi et al., 2008), downregulate Runx2 expression and inhibit osteogenic differentiation of VSMC (Zhu et al., 2013). Moreover, AMPK activator metformin have been shown to reduce coronary and limb arterial calcification in clinical practice (Mary et al., 2017; Lu et al., 2019). All these studies suggested that empagliflozin may inhibit vascular calcification via AMPK signaling pathway. However, the detailed mechanisms still warrant further investigation.

## 5 Conclusion

In conclusion, we have demonstrated that empagliflozin is a novel inhibitor of atherosclerotic calcification *in vivo* and it also inhibits the calcification of VSMCs and aortic rings *in vitro*. Empagliflozin inhibits vascular calcification by inhibiting osteogenic differentiation of VSMCs. Our findings may provide the possibility to meet the clinical need for pharmacological treatment of atherosclerotic calcification.

## Data Availability

All data presented in this article are available by the corresponding author. The raw data of RNA sequencing in this article had been uploaded to the GEO database (GSE243752).
