# Supplementary material for: Empagliflozin alleviates atherosclerotic calcification by inhibiting osteogenic differentiation of vascular smooth muscle cells
Source: Front Pharmacol. 2023 Nov 29;14:1295463. doi: 10.3389/fphar.2023.1295463 (PMC10716287; doi:10.3389/fphar.2023.1295463)
Supplement: Supplementary file 1 [file Table1.docx]

**Supplementary materials**

**Table S1. Sequences of primers used for Rat**

| **Primer name** |  | **Sequence (5' to3')** |
| --- | --- | --- |
| GAPDH | Forward Primer | TTGTGCAGTGCCAGCCTC |
|  | Reverse Primer | GAGAAGGCAGCCCTGGTAAC |
| α-SMA | Forward Primer | CATCCGACCTTGCTAACGGA |
|  | Reverse Primer | AGAGTCCAGCACAATACCAGT |
| SM22-α | Forward Primer | ATCCTATGGCATGAGCCGTG |
|  | Reverse Primer | CCAACTTGCTCAGAATCACGC |
| Smoothelin | Forward Primer | GTGCCCTGGTGCACAATTTC |
|  | Reverse Primer | GTCCGCATGGGTCTCAGC |
| Cnn1 | Forward Primer | ATTGGCCTACAGATGGGCAC |
|  | Reverse Primer | TCAAAGATCTGCCGCTTGGT |
| Runx2 | Forward Primer | GCCAGGTTCAACGATCTGAG |
|  | Reverse Primer | GAGGCGGTVAGAAACAAAC |
| BMP2 | Forward Primer | GACTGCGGTCTCCTAAAGGTCG |
|  | Reverse Primer | CTGGGGAAGCAGCAACACTA |

**Note:** α-SMA: alpha-smooth muscle actin; BMP2: Bone morphogenetic protein 2; Cnn1: calponin 1; Runx2: runt-related transcription factor 2; SM22-α: smooth muscle 22 alpha.
